# Face Mask Usage among Young Polish People during the COVID-19 Epidemic—An Evolving Scenario

**DOI:** 10.3390/healthcare9060638

**Published:** 2021-05-27

**Authors:** Radomir Reszke, Marta Szepietowska, Piotr K. Krajewski, Łukasz Matusiak, Rafał Białynicki-Birula, Jacek C. Szepietowski

**Affiliations:** 1 Department of Dermatology, Venereology and Allergology, Wrocław Medical University, 50-368 Wrocław, Poland; radomir.reszke@umed.wroc.pl (R.R.); piokrzykrajewski@gmail.com (P.K.K.); luke71@interia.pl (Ł.M.); rafal.bialynicki-birula@umed.wroc.pl (R.B.-B.); 2 Students’ Research Group of Experimental Dermatology, Department of Dermatology, Venereology and Allergology, Wrocław Medical University, 50-368 Wrocław, Poland; marta.szepietowska@student.umed.wroc.pl

**Keywords:** face masks, COVID-19, young people, behaviors

## Abstract

The usage of face masks has been mandated in many countries in an attempt to diminish the spread of SARS-CoV-2. In this cross-sectional study, we aimed to determine face mask-wearing behaviors and practices in 1173 young Polish people during the second wave of the COVID-19 epidemic in October 2020. The majority of respondents (97.4%) declared that they wore face masks in areas/situations where it is mandatory. The most common types of utilized face masks were cloth masks (47.7%) and surgical masks (47%), followed by respirators (N95/FFP3) (3.2%) and half-face elastomeric respirators (0.9%). Over 38% reported frequently disinfecting their face masks, especially females. Respondents reporting personal atopic predisposition (64.5% vs. 72.1%; *p* = 0.02) or sensitive skin (65.5% vs. 74.3%; *p* = 0.005) declared multiple use of face masks less commonly than other individuals. Individuals suffering from facial skin lesions declared disinfecting face masks more commonly (40.8% vs. 34.9%; *p* = 0.04). Overall, the self-declared utilization of face masks among young people in Poland has improved since the beginning of the epidemic as compared with our previous study. Until the mass vaccination of the public is achieved and government policy is changed, face mask use remains a valuable tool to decrease the transmission of SARS-CoV-2.

## 1. Introduction

Since the beginning of the COVID-19 pandemic, all countries have been subjected to a situation largely unknown previously. Over a year since pandemic onset (as of 26 April 2021), the number of confirmed cases of SARS-CoV-2 infection exceeds 146.8 million worldwide, with over 3.1 million confirmed deaths [1]. Concurrently, the epidemic situation remained concerning throughout the entire European Union. For example, the total number of confirmed COVID-19 cases in Poland has exceeded 2.7 million (26 April 2021), claiming at least 65,000 officially reported deaths [2]. Owing to the dominance of airborne transmission of SARS-CoV-2 between humans [3] and unavailability of a specific vaccine, prophylactic safety precautions instigated by each individual, endorsed and verified by relevant official services, constituted the crucial step to decrease the development of the pandemic in 2020. The World Health Organization (WHO) and European Centre for Disease Prevention and Control (ECDC) recommendations were aimed at the general public and focused on social distancing, hand hygiene, and usage of personal protective equipment (PPE) [4,5]. These recommendations were also reflected in the official regulations issued by the Polish Government [6,7], which forbade certain activities (e.g., social gatherings, running hotel or restaurant businesses), while particular behaviors became mandatory. An example of the latter is the obligation to strictly cover the nose and the mouth either with face masks or alternatively with a piece of clothing in certain places and routine everyday situations. These include workplaces, public transport, streets, shops, or churches. There is some evidence that the use of face masks may contribute to the prevention of SARS-CoV-2 spread [8,9]. According to Leffler et al. [10], in countries with cultural norms or official regulations which supported face mask-wearing, the COVID-19 epidemic resulted in an average per-capita mortality increase by 16.2% each week. In contrast, other countries experienced a 61.9% weekly mortality increase. More frequent usage of face masks during the COVID-19 pandemic is associated not only with better physical, but also mental health in terms of anxiety, depression, and stress, as recounted in a study comparing Chinese and Polish respondents [11].

Despite the literature evidence and official regulations, these factors still cannot guarantee that each person will continue to wear face masks during the pandemic. Unsurprisingly, there have been significant differences in mask-wearing behaviors and practices during the COVID-19 pandemic in the general public [12,13,14,15]. It seems that certain factors, such as age, sex, education, or dwelling location might affect the face mask-wearing prevalence and/or type of mask used. The possible associations between age and face mask-wearing behaviors are particularly interesting. Firstly, certain studies reported that younger age is related to diminished compliance with face mask-wearing recommendations [13,14,16]. There are multiple causes of this observation, such as certain common inconveniences (e.g., breathing difficulties, sweating, misting of the glasses) [17] which can occur regardless of age. Unfortunately, some individuals regard obligatory face mask usage as a restriction of personal freedom and unethical [18,19]. Secondly, younger people infected with SARS-CoV-2 are relatively more prone to experience an asymptomatic course of COVID-19 [20,21]. Consequently, they do not actively seek medical advice and continue with their usual everyday activities. Additionally, the phenomenon of superspreading deserves particular attention as well. A superspreading event (SSE) refers to a situation in which particular individuals can infect an unusually high number of secondary cases, as reviewed by Lloyd-Smith et al. [22]. The SSE seems to play an important role in the transmission of SARS-CoV-2 [23,24,25]. In a study based on contact tracing, it was revealed that 19% of all detected cases were in fact responsible for 80% of the entire local transmission of SARS-CoV-2 [23]. Epidemiological data from the USA support that SARS-CoV-2 superspreading might also be associated with age. The group of non-elderly (<60 years) subjects with COVID-19 was 2.78 times more likely to spread the infection than the elderly [24], whereas at least 65 out of 100 SARS-CoV-2 infections originated from people aged 20–49 [25]. Such tendencies among age groups may be explained by different associated factors, e.g., occupation or lifestyle.

Notably, due to the prolonged and evolving nature of the COVID-19 pandemic, different epidemiological regulations and restrictions were issued by the authorities. As they changed over time, this could have stimulated confusion and impeded compliance [26]. The dynamic course of the epidemic situation could have also contributed to a scenario in which certain attitudes and behaviors of the general public regarding the use of PPE have also changed over time. Therefore, our study aimed to evaluate the prevalence and detailed characteristics of face mask-wearing behaviors and practices, focusing on young Polish people during the second wave of COVID-19, seven months after the first confirmed case in Poland.

## 2. Materials & Methods

Our study was conducted using an original online survey developed in Google Forms^®^ and subsequently sent to young Polish people, mainly students. Participation in the study was voluntary, with WhatsApp^®^, Facebook^®^, e-mail, or SMS invitation containing a direct link to the questionnaire. Based on the snowball sampling technique [27], each participant was able to send the link further and invite additional participants. We have gathered demographic data, as well as detailed characteristics of self-reported face mask-wearing behaviors and practices, including the type of face masks used, disinfection practices, multiple uses of face masks, as well as the personal history of atopic predisposition, sensitive skin, and facial skin lesions. We intended to perform statistical analysis with a particular emphasis on sex and the presence of skin-related conditions, as previous studies revealed their associations with face mask-wearing practices and behaviors [13,17,28,29]. Data collection occurred between 1–7 October 2020, during the second wave of the COVID-19 epidemic in Poland. The chosen time period was intentional, as it directly preceded the portended reintroduction of official government restrictions and regulations on 9 October 2020 [7]. Prior to those, it had been necessary to wear face masks in closed spaces (e.g., shops), whereas in open spaces (e.g., streets) it had been voluntary, although recommended. Since the introduction of the regulations on October 9th, strict face covering was mandated, either with a face mask or cloth, in various locations and situations in public space. These included public transport, streets, squares, cemeteries, promenades, boulevards, parking lots, and a variety of buildings (offices, schools, banks, shops, healthcare facilities, churches). In total, 1173 individuals aged 20.9 ± 2.9 years (mean ± standard deviation [SD]; range 17–27 years) provided data for further analysis. The number of included participants provided a confidence level of 95%, with a 2.86% margin of error. The majority of respondents in our cohort were females (74.7% vs. 25.3%). Statistical analysis was performed with Statistica 13 software (Dell, Inc, Round Rock, TX, USA) using the Chi-square test. A *p*-value less than 0.05 was considered as statistically significant. The study was performed based on the statutory activity of the department, in accordance with the ethical approval of the Wrocław Medical University Institutional Review Board (ST.C260.18.019).

## 3. Results

### 3.1. Basic Results

The vast majority of our cohort (97.4%) declared that they “often” or “always” wear face masks in areas/situations where it was mandatory. This tendency was also more frequent among females (98.3% vs. 95%; *p* = 0.002). On the other hand, 23% declared that they wear face masks “often” or “always” in areas or situations where it is not mandatory. The most common types of used face masks were cloth (47.7%) and surgical masks (47%), followed by respirators (N95/FFP3) (3.2%) and half-face elastomeric respirators (0.9%) (Table 1). Multiple-use face masks were used by 24.9% of subjects, while 69.6% declared that they use their face masks multiple times (regardless of whether the mask is designed to do so or not). Over one-third (38.3%) reported that they regularly disinfected their face masks, especially females (40.1% vs. 33%; *p* = 0.03). Regarding skin-related conditions reported by our cohort, personal atopic predisposition concerned 33.8%, with a similar prevalence in both sexes. Furthermore, sensitive skin (52.3%) and current presence of facial skin lesions (57.5%) were both reported more commonly by females (60.7% vs. 26.9%; *p* < 0.0001; and 62.6% vs. 42.4%; *p* < 0.0001; respectively).

### 3.2. Skin-Related Conditions and Their Influence on the Type of Face Masks Used and Face Mask-Related Behaviours

Individuals with personal atopic predisposition were less likely than their healthy peers to use face masks multiple times (64.5% vs. 72.1%; *p* = 0.02) (Table 2). Similarly, multiple uses of face masks were also less common among those with sensitive skin (65.5% vs. 74.3%; *p* = 0.005) (Table 3). Additionally, individuals who reported the current presence of facial skin lesions were more prone to disinfect face masks than those with healthy skin (40.8% vs. 34.9%; *p* = 0.04) (Table 4). The presence or absence of skin-related conditions did not favor our participants’ habit of wearing masks in areas/situations in which their use was mandatory or not. Personal atopic predisposition (*p* = 0.26), sensitive skin (*p* = 0.98) and facial skin lesions (*p* = 0.58) did not seem to influence the participants’ choice of particular type of face mask.

## 4. Discussion

It seems reasonable to expect that in countries with a cultural habit of wearing face masks (e.g., Japan) [30], this behavior would be easier to achieve during the ongoing COVID-19 pandemic. Conversely, and despite the epidemic situation, it cannot be ensured that the general public in other countries would adhere to the official mandatory regulations, even when the lack of compliance may result in penalties such as being fined by police or sanitary inspectors. The discussion on personal safety behaviors of the general population may focus on different baseline aspects, with age being one of the most important. Luo et al. [31] postulated that there is a generational gap in terms of undertaking preventive measures recommended by the CDC against SARS-CoV-2, with elderly individuals being more prone to abide by them. There are multiple possible explanations, ranging from higher risk of hospitalization, severe course, and fatal outcome of COVID-19 in the elderly [13,32] to more common adherence to the social norms in their area of residence [33]. Unsurprisingly, some publications revealed that wearing face masks during the COVID-19 pandemic is insufficient among young adults [13,16,28,33,34,35]. As an example, an American study by Haischer et al. [13] reported that among 5517 individuals entering shops in the state of Wisconsin, only 41.5% wore masks. When accounting for age groups, younger subjects (2–30 years old) wore face masks less commonly (37%) than middle-aged (30–65 years) (41%) or elderly (>65 years) (57%). Adjusted odds ratio (aOR) for a middle-aged wearing a face mask was 1.597 higher than for younger individuals (95% confidence interval [CI] 1.359–1.877), while the proportion was even higher when comparing elderly vs. younger individuals (aOR 3.434; 95% CI 2.811–4.195). Likewise, a study conducted on the Spanish population revealed that young individuals (aged 18–25 years) were least likely to wear face masks when compared to all the other age groups [33]. Consequently, in our previous study, we assessed the face mask usage prevalence and mask-related behaviors and practices, deciding to strictly focus attention on young Polish people [12]. The latter study was conducted during the first COVID-19 wave in Poland in April 2020, shortly before the introduction of the first official governmental policy which included mandatory face mask-wearing in public [6]. Therein, out of 2307 young individuals, only 60.4% admitted to wearing face masks, regardless of sex [12]. In contrast, the current study revealed that 97.4% of young respondents wore face masks in mandatory areas/situations during the second COVID-19 wave in October 2020. Although the cohorts were different in both studies and do not justify performing a direct comparison, there was a tendency for higher adherence to the safety regulations throughout the course of the epidemic in Poland. Strzelecki et al. [36] observed a correlation between the spread of the COVID-19 epidemic in Poland and Google Trends searches on PPE, including face masks. We deem these results as important evidence that, as the epidemic develops, people actively broaden their knowledge by seeking health- or life-preserving solutions, as the first step to more frequent and successful usage of PPE. They are also in line with the dynamics of our current and past [12] observations that a change in health-preserving behaviors is a process that occurs over time. Interestingly, the current study revealed that females were more compliant with the face mask-wearing regime (98.3% vs. 95.0%; *p* = 0.002), confirming the findings of previous studies [13,28]. In the setting of an epidemic, women have a crucial role in promoting preventive behaviors among their family members and social community [37]. Notably, 23% of participants also reported that they wear face masks in areas/situations where it is not mandatory. Such careful behavior may be beneficial in certain situations, e.g., when both healthy and COVID-19 positive households wear face masks. Thereby, as reported by Chinese investigators, SARS-CoV-2 transmission to other family members may be reduced by 79%. However, Wang et al. [9] noted that the primary cases needed to wear face masks before the onset of symptoms, whereas later introduction did not seem to play a protective role.

We determined that young people mostly used cloth face masks (47.7%), closely followed by surgical face masks (47%), while respirators (3.2%) and half-face elastomeric respirators (0.9%) were worn less commonly. Still, over half of our cohort utilized face masks with better protective properties (filtration efficacy) than cloth masks [38,39]. In our previous study [12], cloth face masks were also the most frequently used modality (46.2%), while surgical masks (39%) were employed relatively less commonly. The changes in cloth vs. surgical mask usage over time might stem from the fact that, during the initial stages of the epidemic, there was a shortage of surgical masks supply, making it necessary to rely on cloth masks, which are also easier to manufacture. On the other hand, the use of respirators in October 2020 (4.1% in total) was much lower than in April 2020 (14.1% in total). With a more serious epidemic scenario later in 2020, we would expect the general public to use gear with better protection properties, especially acknowledging its better availability over time. Nevertheless, due to their cost, respirators might be in fact less suitable for the general public to use daily. Regarding the impact of baseline skin conditions on face mask-wearing behaviors, the participants of our study with an atopic predisposition or sensitive skin were less prone to use face masks multiple times. Several recent studies have proven the association between face mask usage during the COVID-19 pandemic and diverse cutaneous problems [12,40,41], including the exacerbation of atopic dermatitis predominantly in mask-covered areas [42]. Multiple use of a single face mask could be associated with friction, warmth, and moisture. Additionally, the presence of formaldehyde and other preservatives could predispose to contact dermatitis [41]. These tendencies may be more pronounced, especially in a person with an atopic predisposition or sensitive skin. We also theorize that people with such predispositions (essentially of chronic nature) may possess higher knowledge on proper health-related behaviors and consequently follow the recommendations more thoroughly. Similarly, the current presence of facial skin lesions in our cohort was associated with the more common practice of face mask disinfection. This procedure was undertaken more frequently by females and seems in accordance with a higher prevalence of sensitive skin and current facial skin lesions. People with active inflammatory skin lesions may regard their nature as purely infectious and put more emphasis on hygiene. Conversely, the use of certain chemical disinfectants could predispose to the development of allergic contact dermatitis or contact urticaria [43] and further reinforce the appearance of facial skin lesions.

Our study has several limitations. The design resulted in the self-reported nature of the data acquired from the respondents. Therefore, it is unknown whether all the participants responded to all the questions truthfully. Moreover, a recall bias could also impact the results. Due to the chosen methodology, it is impossible to determine the true response rate. Notwithstanding the frequent declaration of face mask-wearing by the young people participating in this study, it must be noted that the general public and even healthcare workers may not comply with the guidelines on proper utilization of face masks according to the WHO guidelines [29,30]. Therefore, even a high proportion of respondents assessed in a dichotomous manner (wearing vs. not wearing a mask) may not actually benefit from their seemingly protective behavior. Furthermore, despite the rationale of this study explained in precedent paragraphs, a minor limitation might stem from the study concept itself. Essentially, there are conflicting reports in the literature, some of them undermining the basic rationale of the study. As an example, in the only randomized controlled trial assessing face mask-wearing in the community (DANMASK-19), the implementation of surgical masks did not result in a significantly decreased risk of SARS-CoV-2 acquisition [44]. According to ECDC, the effectiveness of medical face masks in preventing COVID-19 in the community is small to moderate, with the certainty of the recommendation being low to moderate [45]. Additionally, our assessment of face mask-wearing behaviors strictly within the particular age group might be debatable. Despite the quoted evidence regarding inadequacies in face mask-wearing behaviors among young people [13,16,28,33,34,35], Howard [15] observed that it is in fact the older individuals who are slightly less likely to wear face masks. We infer that face mask-wearing is not only associated with age, as other cultural and social aspects have to be considered as well. Obviously, due to the young age of the participants, the results of our study cannot be extrapolated to the general public; the situation in other countries, especially those outside of Europe, may also differ. Finally, it is vital to avoid superficial and literal conclusions that could potentially cause harmful accusations and stigmatization towards any fraction of society.

Future evaluations on the use of face masks in the general population should ideally include other age groups from different geographic regions, while the method of assessing face mask-wearing behaviors should more be objective, perhaps by utilization of external observers. In the light of more vaccinated individuals, it would be interesting to determine if the continuous usage of face masks in mostly vaccinated societies could still contribute to the prevention of further SARS-CoV-2 spread. However, it is hoped that with mass anti-COVID-19 vaccination, there will ultimately be no need for mandatory wearing of face masks, at least in certain situations [46]. Therefore, despite the scientific value and potential influence on public health policy, even healthcare professionals may not necessarily anticipate a vast influx of such reports in the near future. Lastly, setting aside the definite eradication of SARS-CoV-2, it is unknown whether another pathogen, possibly of animal origins [47,48,49,50,51], will eventually emerge to reenact the pandemic scenario in the following years or decades, with mandatory re-masking yet again.

## 5. Conclusions

In the light of the second COVID-19 wave in October 2020, the majority of young people in Poland declared that they regularly utilized face masks, as required by official government regulations. Almost half of the respondents utilized cloth masks, closely followed by surgical masks, whereas respirators were reported rarely. Female sex was associated with a higher reported prevalence of sensitive skin and current facial skin lesions. More than one-third of the respondents utilized face mask disinfectants. Females were more likely to perform this action; when compared to males, they were also more prone to wear face masks in areas/situations where it is mandatory. In comparison to our previous study on face mask-wearing behaviors in Poland which was performed in April 2020, it seems that half a year later young people followed the recommendations more meticulously, possibly as a consequence of a more serious epidemic situation and improved awareness of safety behaviors.

## Figures and Tables

**Table 1:** The basic results of the entire cohort according to sex. The *p*-values in bold were considered statistically significant.

Characteristics	Entire Cohort	Females	Males	*p*-Value
N (%)	1173	876 (74.7%)	297 (25.3%)	-
Age (mean ± SD) (years)	20.9 ± 2.9	20.9 ± 2.0	21.2 ± 4.5	*p* = 0.65
**Type of face mask used**				
Cloth	560 (47.7%)	426 (48.6%)	134 (45.1%)	*p* = 0.29
Surgical	552 (47.0%)	409 (46.7%)	143 (48.1%)	*p* = 0.66
Respirator (N95/FFP3)	37 (3.2%)	25 (2.9%)	12 (4.0%)	*p* = 0.31
Half-face elastomeric respirator	10 (0.9%)	6 (0.7%)	4 (1.4%)	*p* = 0.28
None	14 (1.2%)	10 (1.1%)	4 (1.4%)	*p* = 0.79
Only multiple-use face masks	292 (24.9%)	225 (25.7%)	67 (22.6%)	*p* = 0.28
**Behaviors associated with face mask use**				
Face masks worn in areas/situations where it is mandatory (“often” or “always”)	1143 (97.4%)	861 (98.3%)	282 (95.0%)	***p* = 0.002**
Face masks worn in areas/situations where it is not mandatory (“often” or “always”)	270 (23.0%)	192 (21.9%)	78 (26.3%)	*p* = 0.12
Disinfection	449 (38.3%)	351 (40.1%)	98 (33.0%)	***p* = 0.03**
Multiple use of face masks	613 (69.6%)	445 (68.4%)	168 (73.0%)	*p* = 0.18
**Skin-related conditions**				
Personal atopic predisposition	396 (33.8%)	309 (35.3%)	87 (29.3%)	*p* = 0.06
Sensitive skin	613 (52.3%)	532 (60.7%)	81 (26.9%)	***p* < 0.0001**
Current facial skin lesions	674 (57.5%)	548 (62.6%)	126 (42.4%)	***p* < 0.0001**

**Table 2:** The influence of personal atopic predisposition on the type of face mask used and other face mask-related behaviors. The values in bold are considered statistically significant.

Characteristics	Atopic Predisposition (*n* = 396)	No Atopic Predisposition (*n* = 777)	*p*-Value
**Type of face mask used**			
Cloth	183 (46.2%)	377 (48.5%)	*p* = 0.45
Surgical	186 (47.0%)	366 (47.1%)	*p* = 0.97
Respirator (N95/FFP3)	18 (4.5%)	19 (2.4%)	*p* = 0.05
Half-face elastomeric respirator	5 (1.3%)	5 (0.7%)	*p* = 0.28
None	4 (1.0%)	10 (1.3%)	*p* = 0.68
Only multiple-use face masks	100 (25.3%)	192 (24.7%)	*p* = 0.84
**Behaviors associated with face mask use**			
Face masks worn in areas/situations where it is mandatory (“often” or “always”)	387 (97.7%)	756 (97.3%)	*p* = 0.66
Face masks worn in areas/situations where it is not mandatory (“often” or “always”)	90 (23.7%)	180 (23.2%)	*p* = 0.87
Disinfection	165 (41.7%)	284 (36.6%)	*p* = 0.09
Multiple use of face masks	191 (64.5%)	422 (72.1%)	***p* = 0.02**

**Table 3:** The influence of sensitive skin on the type of face mask used and other face mask-related behaviors. The values in bold are considered statistically significant.

Characteristics	Sensitive Skin (*n* = 613)	No Sensitive Skin (*n* = 560)	*p*-Value
**Type of face mask used**			
Cloth	292 (47.6%)	268 (47.9%)	*p* = 0.94
Surgical	287 (46.8%)	265 (47.3%)	*p* = 0.86
Respirator (N95/FFP3)	20 (3.3%)	17 (3.0%)	*p* = 0.82
Half-face elastomeric respirator	6 (1.0%)	4 (0.7%)	*p* = 0.62
None	8 (1.3%)	6 (1.1%)	*p* = 0.71
Only multiple-use face masks	140 (22.8%)	152 (27.1%)	*p* = 0.09
**Behaviors associated with face mask use**			
Face masks worn in areas/situations where it is mandatory (“often” or “always”)	597 (97.4%)	546 (97.5%)	*p* = 0.91
Face masks worn in areas/situations where it is not mandatory (“often” or “always”)	155 (25.3%)	115 (20.5%)	*p* = 0.05
Disinfection	248 (40.5%)	201 (35.9%)	*p* = 0.11
Multiple use of face masks	310 (65.5%)	303 (74.3%)	***p* = 0.005**

**Table 4:** The influence of facial skin lesions on the type of face mask used and other face mask-related behaviors. The values in bold are considered statistically significant.

Characteristics	Facial Skin Lesions (*n* = 674)	No Facial Skin Lesions (*n* = 499)	*p*-Value
**Type of face mask used**			
Cloth	326 (48.4%)	234 (46.9%)	*p* = 0.62
Surgical	319 (47.3%)	233 (46.7%)	*p* = 0.83
Respirator (N95/FFP3)	18 (2.7%)	19 (3.8%)	*p* = 0.27
Half-face elastomeric respirator	4 (0.6%)	6 (1.2%)	*p* = 0.26
None	7 (1.0%)	7 (1.4%)	*p* = 0.57
Only multiple-use face masks	173 (25.7%)	119 (23.9%)	*p* = 0.48
**Behaviors associated with face mask use**			
Face masks worn in areas/situations where it is mandatory (“often” or “always”)	660 (97.9%)	483 (96.8%)	*p* = 0.23
Face masks worn in areas/situations where it is not mandatory (“often” or “always”)	162 (24.0%)	108 (21.6%)	*p* = 0.34
Disinfection	275 (40.8%)	174 (34.9%)	***p* = 0.04**
Multiple use of face masks	348 (69.5%)	265 (69.7%)	*p* = 0.93

## Data Availability

The data obtained in this study may be available from the corresponding author upon a reasonable request.
